# mRNA sequencing revealed transcriptional variability of terpene and eugenol synthase genes among distinct oil yielding accessions of *Ocimum sanctum* L.

**DOI:** 10.1038/s41598-026-54178-9

**Published:** 2026-05-23

**Authors:** Sumitra Panda, Suprava Sahoo, Nikita Panda, Basudeba Kar

**Affiliations:** Centre for Biotechnology, Siksha O Anusandhan (Deemed to be University), Kalinga Nagar Ghatikia, Bhubaneswar, 751003 Odisha India

**Keywords:** *O. sanctum*, Transcriptome profiling, Real time polymerase chain reaction (RT-PCR), GC-MS, Gene expression, Biotechnology, Genetics, Molecular biology, Plant sciences

## Abstract

*Ocimum sanctum* one of the important medicinal plants, lacking adequate molecular resources. In this study, whole mRNA sequencing was carried out towards development of molecular resources, especially correlating with terpenoid biosynthesis and expression of such genes in high and low oil yielding accessions of *O. sanctum*. The transcriptome sequencing resulted 333.91 and 373.10 million high-quality reads in OS1 and OS38 respectively. In OS1 out of 31,820 transcripts, 30,217 transcripts were annotated and in OS38 out of 32,564 transcripts total 30546 transcripts were annotated. KEGG pathway analysis identified 25,643 transcripts in OS1 and 26,382 in OS38. GO and KEGG annotations enabled the identification of trait-specific genes, including those involved in terpenoid biosynthesis. These findings were supported by GC–MS analysis, which showed the presence of 33 terpenoid compounds in the essential oils. Further, relative expression analysis of six genes responsible for terpenoid and eugenol biosynthesis were performed using RT-PCR to confirm transcript presence. Germacrene A Synthase in OS1 and 3 S Linalool Synthase in OS38 showed higher expression as compared to other genes. RT-PCR results were interpreted as qualitative expressions. These results provide scientific insights into transcript diversity of terpenoid biosynthesis and serve as a basis for future genetic improvement of *O. sanctum*.

## Introduction

The primary and most varied class of metabolites among the secondary phytochemical elements that plants make are terpenoids, also known as isoprenoids. It is thought that terpenoid metabolites are essential for a variety of processes, such as development and growth, chemical interactions, and defense against biotic and abiotic environmental stresses^1–3^. Transcriptome profiling has become one of the most utilized approaches for the investigation of specific genes and their regulation at the m-RNA level. It generates a bulk amount of contigs which may be considered as an effective approach to detect the functional gene as well as to characterize the pattern of expression. More importantly, it discloses other biological processes which may offer the comprehension of their underlying mechanisms^4^. Similar to other plants in the *Lamiaceae* family, the *O. sanctum* produces and stores volatile compounds in glandular capitate and peltate trichomes, which are found on the surface of the above-ground parts of the plant^5^. The therapeutic uses of *O. sanctum* along with industrial value for aromatic qualities, highlight the significance of an ethnobotanical approach as a promising source of bioactive compounds. Despite of pharmacological and industrial significance, *O. sanctum* has limited molecular data which restricts research on the biosynthetic pathways of key phytochemicals^6^. *O. sanctum* is renowned for its rich content of various phytochemicals, including eugenol, methyl chavicol, linalool, germacrene, and β-caryophyllene, which belong to the phenylpropanoid and terpenoid categories. Transcriptomic resources serve as effective tools for exploring the biosynthesis of valuable terpenoid compounds in plants. The phenylpropanoids are well-documented in the literature, whereas terpenoids or isoprenoids in *O. sanctum* is relatively limited^7,8^. Isoprenoids are synthesized through two coordinated pathways: the 2-C-methyl-d-erythritol 4-phosphate/1-deoxy-d-xylulose 5-phosphate (MEP/DOXP) pathway and the mevalonate (MVA) pathway. Mono-, di-, and tetraterpenoids are primarily biosynthesized via the MEP/DOXP pathway, while triterpenoids and sesquiterpenoids are produced through the MVA pathway. The coordination of the two isoprenoid pathways in the production of different terpenoids is tightly regulated in relation to the biosynthesis of the terpenoid components^7,9–11^. Universally, isopentenyl pyrophosphate (IPP) and dimethylallyl pyrophosphate (DMAPP) are the two precursor molecules for the terpene synthesis. IPP is mainly involved in cytosolic mevalonate (MVA) pathway whereas, DMAPP takes part in methylerythritol phosphate (MEP) pathways^12^. These two pathways collectively regulate the biosynthesis of terpenoids in plants through coordinated expression of multiple genes and enzymes involved in secondary metabolite production^13,14^.

Addressing all these limitations, transcriptome profiling could be useful in mining of trait specific genes involved in various biological and cellular processes. The de novo transcriptome approach was primarily utilized to identify all expressed transcripts in non-model plants that lack high-quality reference genome data. The purpose of this research was to develop molecular resources, especially correlating with genes responsible for terpenoid biosynthesis and expression study of such genes in high and low oil yielding accessions of *O. sanctum* collected from different agroclimatic zones of Odisha.

## Materials and methods

### Sample collection and oil yield estimation

Total forty accessions of *O. sanctum* were collected from diverse agroclimatic zones of Odisha with essential oil yield (v/w) varied from 0.30 ± 0.06% to 1.25 ± 0.02%. Among these, OS1 showed the highest yield (1.25 ± 0.02%), while OS38 has the lowest yield(0.30 ± 0.06%). Accordingly, these two accessions were selected for subsequent transcriptomic analysis. The plant samples of *O. sanctum* (OS1-Voucher specimen number-2597/CBT, OS38- Voucher specimen number-2614/CBT) were collected from Koraput and Anugul. The taxonomist Prof. (Dr.) Pratap Chandra Panda, CBT, SOADU, Bhubaneswar, Odisha, (Ex-Principal Scientist, Taxonomy and Conservation Division, Regional Plant Resource Centre, Govt. of Odisha, Bhubaneswar) authenticated the species and identified them. The voucher specimen numbers were deposited in the herbarium of Centre for biotechnology, SOA Deemed to be University.

*O. sanctum* accessions OS1 and OS38 were maintained under climatic control greenhouse conditions at Centre for Biotechnology, Siksha ‘O’ Anusandhan (Deemed to be University), Odisha. Leaf samples were collected from plants at the same vegetative developmental stage (approximately 60 days after transplanting), prior to flowering, using fully expanded young leaves from comparable nodal positions. The fresh leaf samples were taken for RNA sequencing.

*O. sanctum* leaves were collected and prepared for extraction of essential oil. Specifically, 100 g of fresh leaf samples were chopped and hydrodistilled for 4 h using a Clevenger-type apparatus. The essential oil yield experiment has been done in triplicates. Yield data were expressed as mean ± standard deviation (SD). The recovered oil was treated with anhydrous sodium sulphate (Na_2_SO_4_) to remove any residual moisture. the dried essential oil was then stored in glass vials at 4 °C until further examination.

Using the following formula, the essential oil yield (fresh weight basis) of the samples was determined in percentage:

$$Yield\:of\:Essential\:Oil\:(v/w)=\frac{Volume\:of\:oil\:collected\:(mL)}{Weight\:of\:plant\:material\:(g)}\times\:100$$

### RNA extraction and quality control

*O. sanctum* frozen leaves were subjected to extract RNA using the Qiagen RNeasy mini kit (Cat No. 74106). Tommy micro smash was used to homogenize about 100 mg of frozen leaf tissue that had been transferred to Tommy tubes along with four stainless steel beads. For the last homogenization cycle, 600 µl of Buffer RLT were used. After fully combining the lysate with half of the volume of absolute alcohol, it was fed into RNeasy spin column that was set inside a 2 ml collecting tube. The tubes were centrifuged for one minute at 10,000 rpm, and the flow through was disposed of. The DNase I (Cat No.79254) treatment on column and the ensuing column washes were carried out in accordance with the manufacturer’s instructions. Water that was nuclease-free was used to elute the RNA from the column. Thermo Scientific’s Nanodrop Spectrophotometer was used to measure the concentration and purity of RNA (2000). RNA integrity was evaluated using Tapestation (Agilent). RNA concentration measured with the Q32855 Qubit RNA HS test kit.

### cDNA library preparation

RNA sequencing libraries were generated using the Illumina-compatible NEBNext^®^ UltraTM II Directional RNA Library Prep Kit (New England BioLabs, MA, USA). 200 ng of total RNA were used as the starting material for mRNA separation, which was then followed by fragmentation and priming. First strand synthesis and then second strand synthesis was applied to the primed and fragmented mRNA. To purify the double-stranded cDNA, NEBNext sample purification beads were used. Following the NEBNext^®^ UltraTM II Directional RNA Library Prep methodology, the purified cDNA was end-repaired, adenylated, and ligated to Illumina adapters. Second strand excision was then carried out using USER enzyme at 37 ˚C for 15 min.In transcriptome sequencing, particularly RNA-Seq, universal adapters and indexed adapters play a crucial role in library preparation and sequencing. These adapters contain key structural features such as the P5 and P7 sequences required for bridge amplification, as well as priming sites for sequencing. Both universal and indexed adapters form the base of Illumina sequencing library structure, making them indispensable in transcriptomics^15^. In RNA-Seq, this strategy enables researchers to study gene expression, splicing patterns, and transcript isoforms on a genome-wide scale. According to^16^, the use of double indexing significantly improves data accuracy by minimizing index misassignment. Likewise^17^, emphasized that RNA-Seq, empowered by such adapter strategies, has revolutionized transcriptomic studies by enabling precise and scalable gene expression analysis.

### De novo assembly and sequence clustering

TrimGalore was used to remove adaptor sequences and low-quality bases (< q30) from the raw reads after FastQC was used for quality evaluation and pre-processing. Using a graph-based method, processed reads were put together by rnaSPAdes, a de novo transcriptome assembly tool for RNA-Seq data that works with a variety of species.T he assembled contigs distinctive parameters, such as their N50 length, average length, maximum length, and lowest length, were computed. The subsequent stage of the assembly process involved grouping the assembled transcripts according to sequence similarity using CD-HIT-EST with a 95% similarity threshold between the sequences, which eliminates sequence diversity without reducing redundancy. TransDecoder was used to identify the possible coding areas within assembled transcript sequences as the final stage in the assembly process. Bowtie was used to align each pre-processed read back to the final transcripts. All assembled transcripts coding sequences were used for additional annotation.

### Gene ontology (GO) and pathway analysis

Using the diamond BLAST program, transcripts were functionally annotated based on homology with respect to “Viridi plantae” protein sequences from the Uniport database. With KAASServer, pathway analysis was performed.

### Identification of transcription factor

Transcription factors play crucial roles as activators or repressors in gene expression. To identify transcription factors from the clean transcripts, the PlantTFDB v4.0 tool (http://planttfdb.cbi.pku.edu.cn/) was employed, using its default parameters. Initially, the tool detected CDS regions from the transcripts using EST scan v3.0^18^. Subsequently, these identified CDS regions were used to pinpoint transcription factors belonging to different families. The threshold for identification was determined using bit-score instead of an e-value cut-off in the PlantTFDB tool. This analysis provided valuable insights into the presence of transcription factors and their potential regulatory functions within the transcriptome of *O. sanctum.*

### Simple Sequence Repeats (SSR) identification

With the use of the MISA10 perl script, SSR were found in every transcript sequence. Simple repeats with motif lengths ranging from monomer to hexamer were found using the MISA’s suggested default settings.

### Essential oil extraction and GC-MS analysis of *O. sanctum*

To analyse terpenoid constituents, GC–MS analysis was carried out using Clarus 580, Perkin Elmer GC instrument attached to Clarus SQ 8 S Mass detector. The instrument was installed with Elite-5 column of 30 m × 0.25 mm internal diameter and 0.25 μm film thickness. Helium gas was used as the mobile phase at a flow rate of 1 ml/minute. The oven temperature was set at 60 °C for 01 min and then heated at 3 °C/minute up to 220 °C, with a hold for 7.00 min. The final run time for the whole program was 60 min. The transfer interface and source temperature were set at 250 °C and 150 °C respectively. The mass spectra of the detecting chemical were compared to the NIST mass spectra library (NIST 11), as well as the retention index (RI) calculated using the n-alkanes series (C_8_-C_20_, Sigma Aldrich, USA). Compounds were identified by computer matching their mass fragmentation spectra with the NIST mass spectra library and comparing RI values acquired from the experiment to data given in the literature^19^.

### Relative gene expression by RT PCR

For the experimental validation of putative transcripts associated with terpenoid biosynthesis, real-time PCR (RT-PCR) was conducted. For the RT-PCR analysis, the Powerup^TM^SYBR™ Green QPCR Master mix and 30 ng of cDNA were utilized. The RT-PCR experiments were carried out in the StepOnePlus Thermal Cycler by Applied Biosystem. The PCR protocol commenced with an initial denaturation step at 95 °C for 2 minutes, followed by 40 cycles involving denaturation at 95 °C for 30 s, annealing at 55 °C-60°Cfor 1 min, and extension at 72 °C for 1 min. The annealing temperature varied depending on melting temperature of specific primers. Subsequently, a melt curve analysis was executed to confirm the specificity of the PCR reaction.

For each gene three biological and technical replicates have been taken for data interpretation. Relative gene expression level was calculated by comparative CT method^20^. Delta CT value has been taken for preparation of graph without normalization.

## Result

### Identification of high and low essential oil yielding accessions

Out of forty accessions from ten agroclimatic zones of Odisha, OS1 accession recorded the highest essential oil yield (1.25 ± 0.02%) and OS38 accession showed the lowest essential oil yield (0.30 ± 0.06%). These two accessions (OS1 and OS38) were considered for further whole mRNA sequencing and especially characterizing genes responsible for terpenoid biosynthesis with expression study to understand the transcriptional variability among various genes responsible for terpenoid biosynthesis.

### Denovo assembly and sequence quality control

**Fig. 1:**
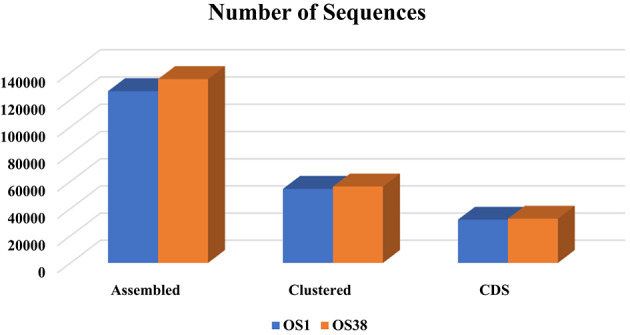
Sequence distribution of high (OS1) and low (OS38) oil yielding accessions of *O. sanctum.*

After sequencing of whole mRNA, all raw reads of OS1 (336.23 million) and OS38 (376.00 million) were subjected to a stringent processing which resulted 333.91 and 373.10 million quality reads in OS1 and OS38accessions respectively. Assembling of quality reads has been done using rnaSPAdes 3.9.1 (Fig. 1) for further annotation study. CD-HIT-EST was used to remove shorter redundant sequences with more than 95% identity and 90% alignment coverage, and non-redundant transcripts were grouped to produce contigs. This was done due to low-quality bases and the existence of adapters, which could impede the assembly process. Total number of contigs obtained from OS1accessionwas 54,498. The average contigs length 1069.18 bp with a maximum length of 16,621 bp and N50 length of 1596 bp. Total number of contigs in OS38 is 56,202. The average contigs length is 1070.97 bp with a maximum length of 16,570 bp and N50 length is 1588 bp.Identification of the coding regions within the assembled transcript sequences was performed as the last step by TransDecoder. A total of 31,820 CDS were obtained from 126,237 number of transcripts in the OS1 accession. The mean CDS length tends to be 934.83 bp with a maximum length of 15,468 bp and minimum length of 297 bp. Similarly, 32,564 number of CDS were obtained from 135,086 transcripts in the OS38 and the mean transcript length tends to be 922.02 bp with a maximum length of 16,113 bp and minimum length of 297 bp. Notably, the N50 value in our study was significantly improved, resulting in the generation of high-quality transcripts given in (Table 1).

**Table 1:** The length distributions of transcripts, clustered and CDS of high (OS1) and low (OS38) oil yielding accessions of *O. sanctum.*

Sequence Length Distribution	OS1			OS38		
	Assembled	Clustered	CDS	Assembled	Clustered	CDS
Number of Sequences	126,237	54,498	31,820	135,086	56,202	32,564
Total Sequences Length	71,877,025	58,268,002	29,746,170	75,207,074	60,190,839	30,024,807
Maximum Sequence Length	16,621	16,621	15,468	16,570	16,570	16,113
Minimum Sequence Length	36	300	297	54	300	297
Average Sequence Length	569.38	1069.18	934.83	556.73	1070.97	922.02
Number of non-ATGC Characters	38,614	38,614	60,913	23,237	23,237	33,187
% of non-ATGC Characters	0.0537	0.0663	0.2048	0.0309	0.0386	0.1105
Sequences < 100 bp	255	0	0	330	0	0
Sequences > = 100–200 bp	40,461	0	0	44,513	0	0
Sequences > = 200–300 bp	30,981	0	56	33,984	0	66
Sequences > = 300–400 bp	11,921	11,893	6436	11,956	11,922	6832
Sequences > = 400–500 bp	6747	6740	4206	7051	7039	4407
Sequences > = 500–600 bp	4912	4907	3036	5184	5177	3181
Sequences > = 600–700 bp	3628	3627	2520	3882	3879	2550
Sequences > = 700–800 bp	2933	2933	1957	3086	3086	1975
Sequences > = 800–900 bp	2442	2441	1602	2555	2554	1724
Sequences > = 900-1Kbp	2046	2046	1674	2159	2159	1620
Sequences > = 1-5Kbp	19,551	19,551	10,215	20,004	20,004	10,091
Sequences > = 5Kbp-1Mbp	376	360	118	382	382	118
n50 Value	1215	1596	1221	1182	1588	1203

### Functional annotation of *O. sanctum* transcripts

**Fig. 2:**
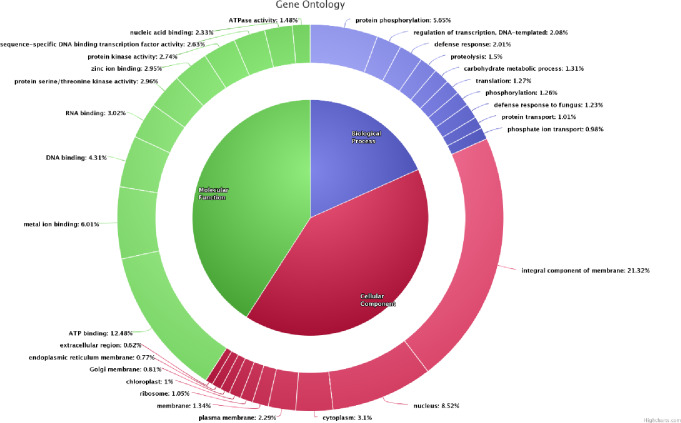
Gene Ontology (GO) annotation of *O. sanctum* (OS1) transcripts linked to molecular function (40.91%), biological Process (18.3%) and cellular components (40.82%) in OS1.

**Fig. 3:**
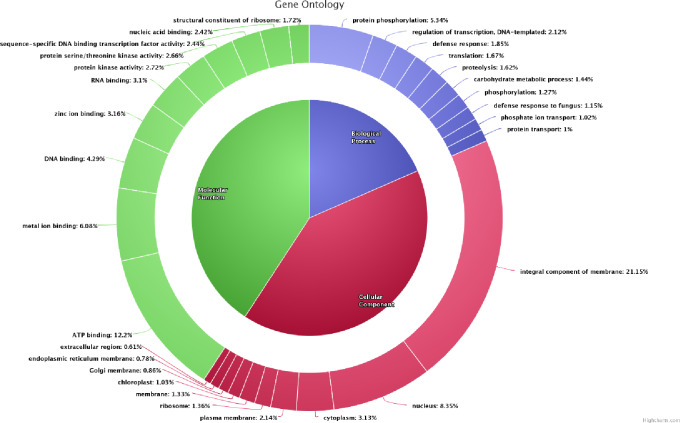
Gene Ontology (GO) annotation of *O. sanctum* (OS38) transcripts linked to molecular function (40.79%), biological Process (18.48%) and cellular component (40.74%) in OS38.

Generally, gene ontology (GO) was used to classify the functions of the assembled transcripts. The results were distributed into three classes such as biological processes, cellular components and molecular functions. The CDS sequences were taken for further analysis. Out of 31,820 transcripts, 30,217 (94.96%) transcripts were annotated functionally and 1603 transcripts were not annotated in OS1accession. Similarly, in OS38accession, out of 32,564 transcripts total annotated transcripts were 30,546 (93.80%) and 2018 transcripts were not annotated. In OS1accession the majority of functions fell into the “molecular function” category, comprising 40.91%, followed by “cellular process” at 40.82%, and “biological processes” at 18.3%. Within the “molecular function” category, the contigs were prominently enriched in terms such as “ATP binding” (12.48%), “metal ion binding” (6.01%),“DNA binding” (4.31%) “RNA binding (3.02%). In the “cellular process” category, the terms “integral component of membrane” (21.32%) and “nucleus” (8.52%) were the most prevalent. In “biological process” category, the predominant matches with protein phosphorylation (5.65%), regulation of transcription, DNA templated (2.08%) and defense response (2.01%) (Fig. 2). Likewise, a similar pattern was found in the OS38 accession, where the “molecular function” category accounted for 40.79%, followed by “cellular process” (40.74%), and “biological processes“(18.48%) (Fig. 3).

**Fig. 4:**
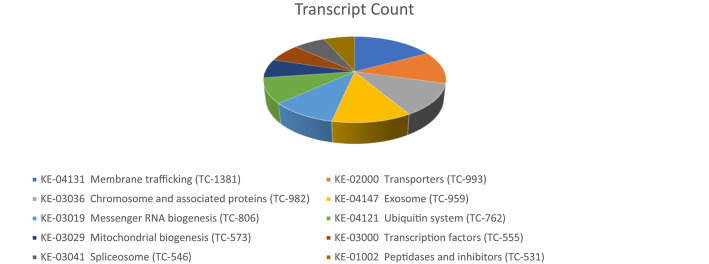
Top ten pathways annotated both with the transcripts of *O. sanctum* (both O1 and OS38) by using Kyoto Encyclopedia of Genes and Genomes (KEGG) database. Membrane trafficking (1381), Transporters (993), Chromosome and associated proteins (982) are having highest transcript counts. Note-KE-KEGG entry number, TC- Transcript count.

KEGG pathway analysis was conducted to identify the active biochemical pathways in OS1 and OS38 accessions. The CDS were taken for further analysis. Out of 31,820 transcripts, 25,643(80.58%) were annotated in OS1 accession and out of 32,564 transcripts, 26,382(81.01%) were annotated in OS38 accession that participate in 202 pathways among OS1 and OS38 accessions respectively. Among these pathways, the top 10 pathways are consistent in both the sample and encompass essential cellular processes such as membrane trafficking, exosome functions, messenger RNA biogenesis, chromosome and associated proteins, transport mechanisms, the ubiquitin system, spliceosome activities, mitochondrial biogenesis, and ribosome biogenesis (Fig. 4). Functional categorization revealed that Membrane trafficking (1381), Transporters (993), Chromosome and associated proteins (982) are having highest transcript counts.

### Genes related to terpenoid biosynthesis in *O. sanctum*

**Fig. 5:**
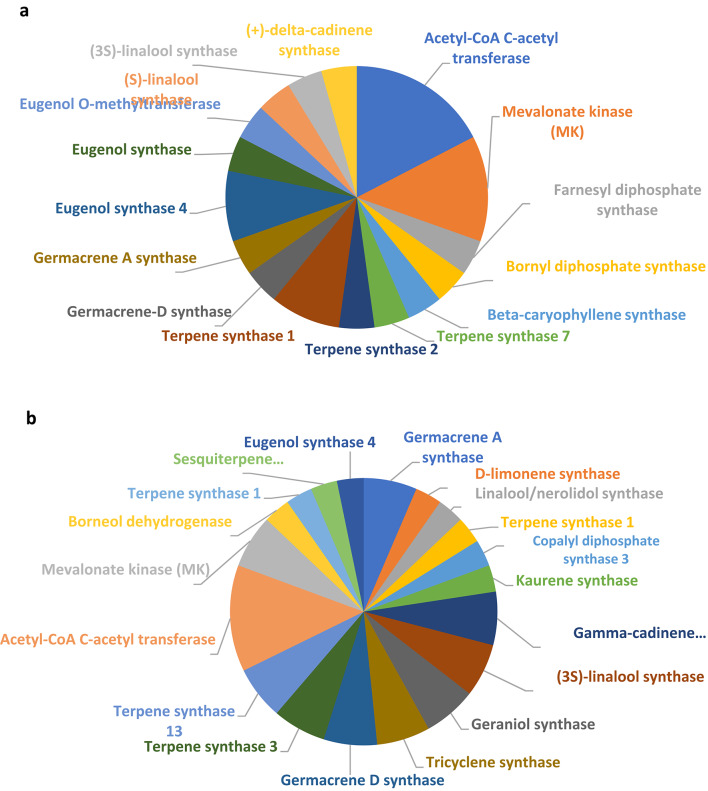
Mining of genes responsible for terpenoid biosynthesis using Gene Ontology: (**a**) In OS1 accession, 23 transcripts were detected, including acetyl-CoA C-acetyl transferase (4), mevalonate kinase (3), and farnesyl diphosphate synthase (1), terpene synthase-1 (2), terpene synthase-2 (1), bornyl diphosphate synthase (1), β-caryophyllene synthase (1), germacrene A (1), germacrene D synthases (1), linalool synthases (1), and δ-cadinene synthase (1), as well as eugenol synthase(4) and eugenol O-methyltransferase (1). (**b**) In OS38 accession, 31 transcripts were identified, including acetyl-CoA C-acetyl transferase (4) and mevalonate kinase (2), terpene synthase-3 (2), terpene synthase-13 (2), germacrene A (1), germacrene D synthases (2), 3s linalool synthase (2), limonene synthase, linalool/nerolidol synthase, geraniol synthase (2), tricyclene synthase (2), and γ-cadinene synthase (2), copalyl diphosphate synthase (1), kaurene synthase (1), borneol dehydrogenase (1), and eugenol synthase (1).

The transcriptomic analysis of OS1 and OS38accessionsrevealed notable differences in terpenoid biosynthesis pathway associations based on GO and KEGG database annotations^21^. According to Gene Ontology (GO) annotations, 23 transcripts in OS1 and 31 in OS38 were primarily linked to terpenoid biosynthetic processes, suggesting a slightly higher representation of functional genes related to this pathway in OS38. In contrast, KEGG pathway mapping identified a significantly greater number of associated transcripts in OS1 (192) compared to OS38 (107), indicating a greater diversity of engagement of terpenoid-related genes in OS1 at the pathway level (Fig. 5a, b).

This discrepancy reflects differences in annotation frameworks: GO classifies genes based on functional categories, while KEGG maps genes to defined metabolic pathways that may include multiple enzymatic steps and isoforms. As a result, KEGG-based analysis captures broader pathway engagement, whereas GO-based annotation highlights specific functional assignments. GO and KEGG analysis enabled to identify trait specific genes especially mining of genes responsible for various terpenoid biosynthesis in high and low oil yielding accessions of *O. sanctum*. Based on this data, key genes responsible for terpenoid biosynthesis have been selected for further relative gene expression study and to unmask scientific mechanisms behind production of volatile compound in the taxa.

**Fig. 6:**
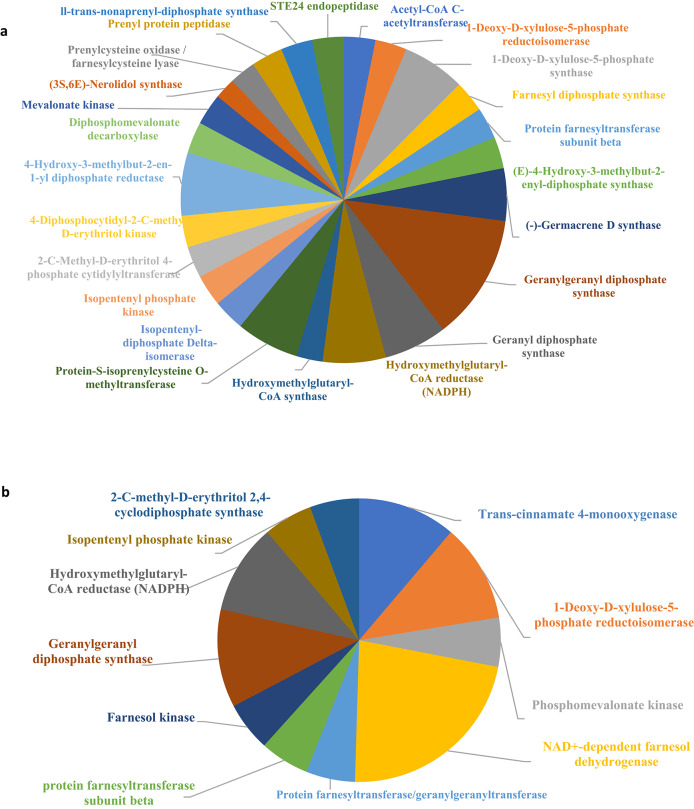
Kyoto Encyclopedia of Genes and Genomes (KEGG) annotation showing transcripts related to terpenoid biosynthesis: (**a**) In OS1 accession, transcripts of 1-Deoxy-D-xylulose-5-phosphate synthase (12), Farnesyl diphosphate synthase (6), Protein farnesyltransferase subunit beta (6), (E)-4-Hydroxy-3-methylbut-2-enyl-diphosphate synthase (6), (−)-Germacrene D synthase (10), Geranylgeranyl diphosphate synthase (24), Geranyl diphosphate synthase (12), Hydroxymethylglutaryl-CoA reductase (NADPH) (12), Hydroxymethylglutaryl-CoA synthase (5), Protein-S-isoprenylcysteine O-methyltransferase (12), Isopentenyl-diphosphate Delta-isomerase (6), Isopentenyl phosphate kinase (6), 2-C-Methyl-D-erythritol 4-phosphate cytidylyltransferase (6), 4-Diphosphocytidyl-2-C-methyl-D-erythritol kinase (6), 4-Hydroxy-3-methylbut-2-en-1-yl diphosphate reductase (12), Diphosphomevalonate decarboxylase (6), Mevalonate kinase (6), (3 S,6E)-Nerolidol synthase (4), Prenylcysteine oxidase / farnesylcysteine lyase (5), Prenyl protein peptidase (6), All-trans-nonaprenyl-diphosphate synthase (6), STE24 endopeptidase (6) were identified. (**b**) In OS38 accession, transcripts of Trans-cinnamate 4-monooxygenase (12), 1-Deoxy-D-xylulose-5-phosphate reductoisomerase (12), Phosphomevalonate kinase (6), NAD⁺-dependent farnesol dehydrogenase (24), Protein farnesyltransferase/geranylgeranyltransferase (6), Protein farnesyltransferase subunit beta (6), Farnesol kinase (6), Geranylgeranyl diphosphate synthase (12), Hydroxymethylglutaryl-CoA reductase (NADPH) (11), Isopentenyl phosphate kinase (6), 2-C-methyl-D-erythritol 2,4-cyclodiphosphate synthase (6) were identified.

Gene Ontology (GO)-based annotation highlighted Acetyl-CoA C-acetyltransferase as a key enzyme in both samples, reflecting its fundamental role in initiating the mevalonate pathway, a central route in terpenoid biosynthesis^22^. Meanwhile, KEGG analysis pointed to different enzymes in the two samples: Geranylgeranyl diphosphate synthase (GGPPS) in OS1 accession, which is involved in the production of precursors for diterpenes and carotenoids, and NAD⁺-dependent farnesol dehydrogenase in OS38 accession, which plays a critical role in sesquiterpene biosynthesis (Fig. 6a, b). Gene Ontology (GO) annotation identified transcripts related to terpenoid and phenylpropanoid biosynthesis in both accessions. In OS1, 23 transcripts were detected, including acetyl-CoA C-acetyl transferase (4), mevalonate kinase (3), and farnesyl diphosphate synthase (1) along with several terpene synthases such as bornyl diphosphate synthase, β-caryophyllene synthase, germacrene A, germacrene D synthases, linalool synthases, and δ-cadinene synthase, as well as eugenol synthase and eugenol O-methyltransferase. In OS38, 31 transcripts were identified, including acetyl-CoA C-acetyl transferase (4) and mevalonate kinase (2), along with multiple terpene synthases such as germacrene A, germacrene D synthases, limonene synthase, linalool/nerolidol synthase, geraniol synthase, tricyclene synthase, and γ-cadinene synthase, in addition to copalyl diphosphate synthase, kaurene synthase, borneol dehydrogenase, and eugenol synthase. These results indicate the presence of a diverse set of genes involved in volatile terpenoid biosynthesis in both accessions.

KEGG pathway annotation identified multiple enzymes involved in terpenoid backbone biosynthesis in both accessions. In OS1 accession, key enzymes included acetyl-CoA C-acetyltransferase (6), 1-deoxy-D-xylulose-5-phosphate synthase (12), DXR (6), farnesyl diphosphate synthase (6), geranyl diphosphate synthase (12), and geranylgeranyl diphosphate synthase (24), along with terpene synthases such as germacrene D synthase (10) and nerolidol synthase (4). In OS38 accession, enzymes of the MEP and MVA pathways such as DXR (12), MECDP synthase (6), phosphomevalonate kinase (6), and HMG-CoA reductase (11) were identified, together with geranylgeranyl diphosphate synthase (12) and modification enzymes including farnesol dehydrogenase (24) and farnesol kinase (6). These results indicate active terpenoid biosynthetic pathways in both accessions.

Comparative pathway analysis indicates that OS1 accession exhibits representation of backbone-generating enzymes, particularly geranylgeranyl diphosphate synthase and geranyl diphosphate synthase, suggesting possible differences in precursor allocation towards mono-, sesqui-, and diterpenoid biosynthesis. In contrast, OS38 accession represents NAD⁺-dependent farnesol dehydrogenase and related modification enzymes, implying preferential downstream diversification of sesquiterpene alcohols. his representation may indicate accession-specific transcript distribution rather than uniform upregulation of the entire terpenoid pathway. These observations represent differences in transcript presence and annotation patterns and should not be interpreted as statistically supported differential expression.

### Transcription factor regulating terpenoid biosynthesis

**Fig. 7:**
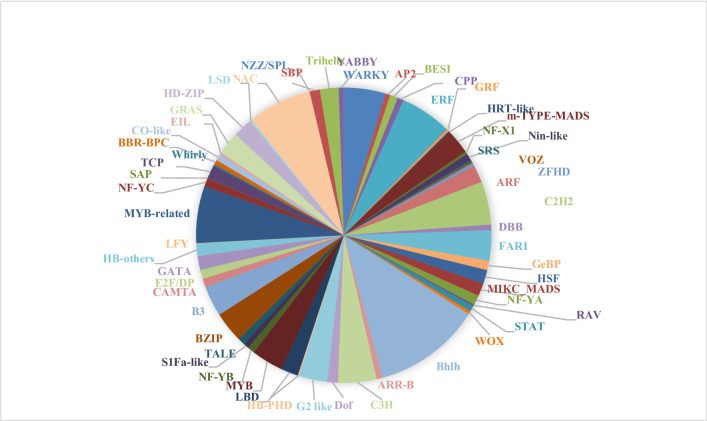
Identified transcription factors from *O. sanctum* transcripts involved in regulating various gene expression.

**Fig. 8:**
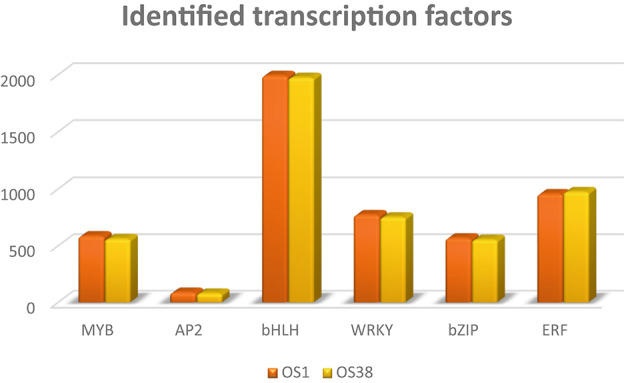
Identified transcription factors from *O. sanctum* accessions (OS1 and OS38) involved in terpenoid biosynthesis.

Transcription factors (TFs) are key regulatory proteins that control gene expression and play crucial roles in various physiological and metabolic processes in plants^23^. In the present study, transcription factors were identified from the *O. sanctum* transcriptome using the Plant Transcription Factor Database (PlantTFDB). A total of 58 different TF families were predicted from the transcripts (Fig. 7), reflecting the complex regulatory network present in this medicinal plant. Among these, specific transcription factor families known to regulate terpenoid biosynthesis were examined in detail. These include MYB, AP2, bHLH, WRKY, bZIP, and ERF, all of which are known to influence secondary metabolite pathways, including terpenoid production (Fig. 8). These transcription factors can down regulate and upregulate the gene expression by binding to the promoter regions^24^. Cytochrome P450 has also very important role in terpenoid biosynthesis. From the GO-annotation dataset, 47 Cytochrome P450 genes were recognised belonging to different families.

### Identification of quality SSR

MISA tool was used to find SSRs motifs from the clean transcripts of *O. sanctum*. MISA tool examined 31,110 number of sequences having total size 29,391,015 bp. A total of 2,958 SSRs were identified in OS1, while 3,065 SSRs were identified in OS38.Large number of SSR were found in trinucleotide repeat (1441) followed by dinucleotide repeats (603), tetranucleotide repeats (514), mononucleotide repeats (359), hexanucleotide repeats (37), and pentanucleotide repeats (5) in OS1 accession.

**Fig. 9:**
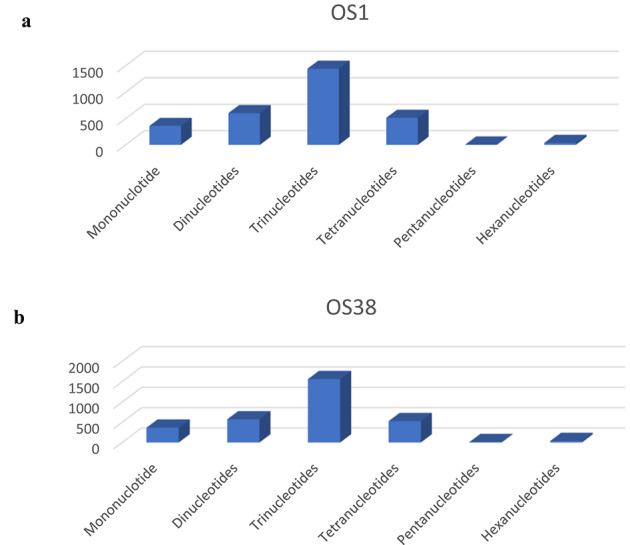
Distribution of different identified Simple sequence repeats SSRs in *O. sanctum* accessions. (**a**) identified Simple sequence repeats SSRs in OS1 and (**b**) identified Simple sequence repeats SSRs in OS38.

Whereas in OS38 accession, trinucleotide repeats (1559) are more followed by dinucleotide repeats (571), tetranucleotide repeats (527), mononucleotide repeats (196), hexanucleotide repeats (36), and pentanucleotide repeats (9) (Fig. 9). The identified SSRs are summarised in (Table 2).Mining of transcript-derived SSRs resulted trinucleotide repeats as the predominant motif class in both the accessions, with differences in SSR abundance which could be a resource for future molecular studies.

**Table 2:** Summary of identified SSRs in transcripts of OS1 and OS38.

Sequence Statistics	OS1	OS38
Total number of sequences examined	31,110	31,807
Total size of examined sequences (bp)	29,391,015	29,639,958
Total number of identified SSRs	2958	3065
Number of SSR containing sequences	2576	2648
Number of sequences containing more than 1 SSR	308	347
Number of compounds SSRs (i.e. c)	202	203
Mononucleotide repeats (p1)	359	364
Dinucleotide repeats (p2)	603	571
Trinucleotide repeats (p3)	1441	1559
Tetranucleotide repeats (p4)	514	527
Pentanucleotide repeats (p5)	5	9
Hexanucleotide repeats (p6)	37	36

### Identification of terpenoid constituents in *O. sanctum*

In this experiment, *O. sanctum* leaves were taken for extraction of essential oil and were analysed using GC-MS. Further the compounds were identified using Adam’s library^19^ and NIST library. In OS1 accession, GC-MS analysis identified 33 constituents and the major terpenoid constituents are eugenol (50.379 ± 0.03%) followed by β-caryophyllene (16.932 ± 0.02%), β-Elemene (15.351 ± 0.04%), Germacrene A (3.458 ± 0.02%), Germacrene D (2.319 ± 0.01%). Similarly, in OS38 accession, GC-MS analysis identified 33 constituents in which the major one is Eugenol (47.922 ± 0.03%) followed by β-Elemene (19.143 ± 0.02%), β-caryophyllene (15.383 ± 0.20%), Germacrene A (3.247 ± 0.02%), Germacrene D (2.180 ± 0.03%). Out of 33 identified constituents, six representative transcripts associated with the biosynthetic pathways of compounds like eugenol synthase, germacrene D synthase, germacrene A synthase, (3 S)-linalool synthase, nerolidol synthase, and γ-cadinene synthase were selected for subsequent gene expression analysis. The details of constituents are given in (Table 3).

**Table 3:** Chemical profile obtained in high (OS1) and low (OS38) oil yielding accessions of *O. sanctum* by gas chromatography and mass spectrometry (GCMS).

SI NO.	Compounds Name	Classification	Ri^a^	Ri^b^	OS1	OS38
1	α-Pinene	Monoterpene hydrocarbon	933	932	0.304 ± 0.01	0.318 ± 0.01
2	Camphene	Monoterpene hydrocarbon	949	946	0.284 ± 0.03	0.286 ± 0.02
3	β-Pinene	Monoterpene hydrocarbon	978	974	-	0.196 ± 0.01
4	Limonene	Monoterpene hydrocarbon	1031	1024	0.089 ± 0.02	-
5	β-Ocimene	Monoterpene hydrocarbon	1046	1044	0.321 ± 0.02	-
6	Linalool	Oxygenated monoterpene	1102	1095	0.160 ± 0.02	0.163 ± 0.02
7	Isoborneol	Oxygenated monoterpene	1172	1155	0.575 ± 0.01	0.635 ± 0.02
8	α-Cubebene	Sesquiterpene hydrocarbon	1347	1348	0.02 ± 0.01	-
9	**Eugenol**	Phenylpropanoid	1368	1355	**50.379 ± 0.03**	**47.922 ± 0.03**
10	α-Copaene	Sesquiterpene hydrocarbon	1374	1374	0.384 ± 0.02	-
11	β-Bourbonene	Sesquiterpene hydrocarbon	1382	1387	0.933 ± 0.02	1.358 ± 0.01
12	β-Elemene	Sesquiterpene hydrocarbon	1393	1389	15.351 ± 0.04	19.143 ± 0.02
13	Methyl eugenol	Phenylpropanoid	1402	1403	0.254 ± 0.02	0.335 ± 0.02
14	β-Caryophyllene	Sesquiterpene hydrocarbon	1422	1417	16.932 ± 0.02	15.383 ± 0.20
15	α-Humulene	Sesquiterpene hydrocarbon	1452	1452	1.216 ± 0.02	1.162 ± 0.01
16	Cadina-1,6,4-diene	Sesquiterpene hydrocarbon	1469	1461	0.178 ± 0.02	-
17	Germacrene-D	Sesquiterpene hydrocarbon	1478	1480	2.319 ± 0.01	2.180 ± 0.03
18	γ-Hemachalene	Sesquiterpene hydrocarbon	1485	1481	0.480 ± 0.02	-
19	β-Selinene	Sesquiterpene hydrocarbon	1492	1489	-	0.475 ± 0.02
20	α-Selinene	Sesquiterpene hydrocarbon	1498	1492	-	0.544 ± 0.02
21	α-Muurolene	Sesquiterpene hydrocarbon	1513	1500	0.1 ± 0.01	-
22	Germacrene A	Sesquiterpene hydrocarbon	1505	1508	3.458 ± 0.02	3.247 ± 0.02
23	γ-Cadinene	Sesquiterpene hydrocarbon	1514	1513	0.261 ± 0.02	0.335 ± 0.02
24	α-cardinene	Sesquiterpene hydrocarbon	1539	1537	-	0.1 ± 0.01
25	Elemol	Oxygenated sesquiterpene	1546	1548	0.538 ± 0.03	0.710 ± 0.02
26	Nerolidol	Oxygenated sesquiterpene	1577	1561	0.566 ± 0.03	0.029 ± 0.02
27	Caryophyllene oxide	Oxygenated sesquiterpene	1588	1582	0.000 ± 0.01	0.698 ± 0.03
28	Curzerenone	Oxygenated sesquiterpene	1596	1605	0.241 ± 0.03	-
29	γ-Eudesmol	Oxygenated sesquiterpene	1630	1630	0.1 ± 0.02	-
30	α-Cardinol	Oxygenated sesquiterpene	1654	1652	0.914 ± 0.01	0.898 ± 0.04
31	Bisabol-11-ol	Oxygenated sesquiterpene	1668	1667	0.254 ± 0.01	-
32	Nerolidyl acetate(ε)	Oxygenated sesquiterpene	1726	1716	-	0.339 ± 0.01
33	Isolongifolol	Oxygenated sesquiterpene	1721	1728	0.285 ± 0.02	-

Note: Ri^a^-Calculated Retention Inex; Ri^b^-Literature Retention Index. Accessions are shown with area % and Mean ± StDev (SD): Note- Data are analysed in triplicates.

### Relative gene expression study

Out of six genes studied, Germacrene D Synthase, Germacrene A Synthase, 3 S-Linalool Synthase, Nerolidol Synthase, and Gamma-Cadinene Synthase involved in terpenoid biosynthesis belong to the Terpene Synthase (TPS) gene family, Whereas Eugenol Synthase (EGS), responsible for eugenol biosynthesis, belongs to the PIP family of NADPH-dependent reductases associated with the phenylpropanoid metabolism. Eugenol synthase coming under PIP family which converts coniferyl acetate to eugenol. The PIP family, which includes eugenol synthase (EGS), stands for pinoresinol-isoflavone-phenyl coumaran (or sometimes referred to as pinoresinol-lariciresinol reductase (PLR), isoflavone reductase (IFR), and phenyl coumaran benzylic ether reductase (PCBER).

**Fig. 10:**
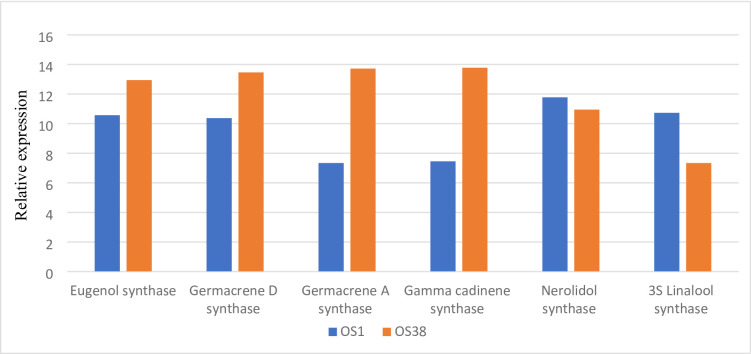
Gene expression of Eugenol Synthase, Germacrene D Synthase, Germacrene A Synthase, 3 S Linalool Synthase, Nerolidol Synthase, and Gamma Cadinene Synthase through RT-PCR in OS1 and OS38. Delta CT value has been taken for graph preparation without normalization.

Comparative relative gene expression study has been done among two high and low oil yielding accessions (OS1 and OS38) with respect to each gene and it was observed that out of six genes studied, four genes such as Eugenol synthase, Germacrene D Synthase, Germacrene A Synthase and Gamma Cadinene Synthase showed higher expression in high oil yielding accession OS1 than low oil yielding accession OS38. Whereas two genes such as 3 S Linalool Synthase and Nerolidol Synthase showed lower expression in OS1 than OS38 (Fig. 10).

Similarly, comparative relative gene expression has also been analyzed in individual accession with respect to each six genes and found that, Germacrene A Synthase showed highest expression in OS1 accession followed by Gamma Cadinene Synthase, Germacrene D Synthase, Eugenol Synthase,3 S Linalool Synthase and Nerolidol Synthase. Whereas in OS38 accession, 3 S Linalool Synthase showed higher expression followed by Nerolidol Synthase, Eugenol Synthase, Germacrene D Synthase, Germacrene A Synthase and Gamma Cadinene Synthase (Fig. 10).

The corresponding terpenoid compounds were also detected in essential oil of *O. sanctum* leaves which were subsequently identified using Gas Chromatography-Mass Spectrometry (GC-MS) analysis. The detected transcripts correspond to metabolites identified through GC–MS analysis, indicating a qualitative alignment between transcript presence and metabolite detection. Relative expression analysis of six key genes was performed using RT-PCR to validate transcript presence. Due to the lack of biological replicates in RNA-Seq libraries, RT-PCR results were interpreted as qualitative expression.

## Discussion

De novo transcriptome assembly refers to the process of reconstructing transcript sequences from RNA-Seq data without a reference genome. It is particularly valuable in non-model organisms like *O. sanctum*, enabling the study of gene expression profiles and terpenoid biosynthesis. In the present study, rnaSPAdes was used for de novo assembly of OS1 and OS38 accessions, producing high-quality assemblies with GC content ranging between 48 and 49%, a parameter indicating genomic stability. The read quality was consistently high, with > Q30 scores, supporting the reliability of the data. Contigs were generated and further processed using CD-HIT to reduce redundancy, followed by CDS prediction via TransDecoder. The assembled transcriptome exhibited high N50 values and robust CDS lengths, signifying a strong assembly suitable for downstream applications in functional and pathway analyses. These findings are in agreement with earlier studies^25^. reported similar GC content and quality metrics in *Ocimum tenuiflorum*, highlighting the gene richness of its transcriptome^26^. also reported successful assembly of *O. basilicum* with high-quality reads^27^. validated the performance of RNA SPAdes in plant transcriptomes, affirming its accuracy and suitability for large-scale assemblies. “Similarly^28^, applied CD-HIT *in O. basilicum* for contigs clustering, and^29^ used TransDecoder” effectively in *Withania somnifera* to identify coding sequences. The current results align well with previous research, reinforcing the robustness and reliability of the applied pipeline for transcriptomic studies in *O. sanctum* and other medicinal plants.

Functional annotation refers to the classification of transcripts into known biological categories such as molecular functions, cellular components, and biological processes, enabling insights into gene activity and physiological roles. In the current study, *O. sanctum* transcripts showed high annotation success, with over 94% of sequences functionally characterized, indicating a reliable and complete transcriptome assembly. The analysis revealed a dominant presence of genes associated with “molecular function,” especially ATP binding (24.68% in OS1 and OS38) and metal ion binding (12.09% in OS1 and OS38), along with transcripts related to membrane-associated structures (16.87% in OS1 and OS38) and nuclear components (42.47% in OS1 and OS38) under the “cellular component” category suggesting active cellular metabolism and regulation in leaf tissue. Additionally, protein phosphorylation (10.99% in OS1 and OS38) and defense response (6.24% in OS1 and OS38) were highly represented within “biological processes,” highlighting their potential roles in signal transduction and plant stress responses. KEGG pathway analysis identified 202 pathways in both OS1 and OS38 samples, covering transport mechanisms, chromatin modification, and spliceosomal pathways. These observations align with previous transcriptomic studies in medicinal plants, where^26^ reported enrichment of transporters, ribosomal processes, and ubiquitin-mediated pathways in *Ocimum* species, while^30^ described membrane trafficking and mRNA processing pathways in *Withania somnifera* and *Mentha arvensis*, respectively^31^. also noted similar pathway enrichment in *O. kilimandscharicum*, suggesting a conserved functional signature across *Ocimum* species. The functional annotation outcomes are consistent with earlier findings, reinforcing the active role of these genes and pathways in maintaining physiological balance and adaptive responses in *O. sanctum* leaf tissues.

Transcription factors (TFs) are proteins that regulate gene expression by binding to specific DNA sequences, playing a pivotal role in controlling various biological processes, including secondary metabolism. In the present study, 58 transcription factor families were identified in the *O. sanctum* transcriptome, reflecting a complex regulatory landscape associated with terpenoid biosynthesis. Major TF families such as MYB, bHLH, AP2/ERF, WRKY, and bZIP were abundantly represented, suggesting their active involvement in modulating the expression of genes linked to specialized metabolic pathways. Notably, 47 Cytochrome P450 genes were also identified, indicating their contribution to the structural diversification of terpenoids. These results underscore the orchestrated interaction between transcriptional regulators and enzymatic components in shaping the terpenoid profile of *O. sanctum*. Similar findings have been reported in other medicinal plants, where MYB and bHLH transcription factors regulate terpenoid and flavonoid biosynthesis in *Arabidopsis thaliana* and *Catharanthus roseus*^32^, while WRKY and ERF transcription factors have been reported to control monoterpene synthesis^33^. Additionally, the role of Cytochrome P450s in terpenoid diversification has been well documented by^22^, supporting their significance in specialized plant metabolism. The current results reinforce the established understanding that transcription factors and Cytochrome P450s together form a regulatory framework essential for secondary metabolite production in *O. sanctum*. Simple sequence repeats (SSRs), also known as microsatellites, are short, tandemly repeated DNA sequences widely used as molecular markers due to their high polymorphism, co-dominant inheritance, and abundance across genomes. In this study, a total of 2,958 SSRs were identified from the *O. sanctum* transcriptome using the MISA tool, demonstrating the rich potential of transcript-derived SSRs (genic-SSRs) for molecular breeding and genetic diversity assessments. The analysis revealed a predominance of trinucleotide repeats, followed by tetra- and dinucleotide motifs, indicating their preferential localization within coding regions. The presence of compound SSRs and sequences with multiple SSR motifs points to regions of high genomic variability, which are valuable for population structure and functional diversity studies. Notably, a higher number of SSRs were detected in the OS38 sample, suggesting genotypic differences in SSR distribution. These observations are in line with previous reports in *O. basilicum*^34^ and *Mentha arvensis*^35^, where trinucleotide SSRs were most abundant due to their compatibility with protein-coding sequences. Similar genotypic variation was also noted in *Withania somnifera* by^36^. The successful mining of SSRs from transcriptomic data further supports earlier findings by^37^, who emphasized the utility of genic-SSRs for marker-assisted selection in medicinal plants. SSRs may serve as potential markers for future genetic diversity studies. Terpenoid biosynthesis refers to the metabolic processes through which plants produce a diverse class of secondary metabolites synthesized via the mevalonate (MVA) and methylerythritol phosphate (MEP) pathways. These compounds play vital roles in plant defense, development, and ecological interactions. In the present study, the differential representation of terpenoid biosynthetic genes between OS1 and OS38 accessions of *O. sanctum* highlights the dynamic and possibly genotype-specific regulation of secondary metabolism. Notably, OS1 transcripts showed the presence of a broader diversity of enzymes, including geranylgeranyl pyrophosphate synthase (GGPPS), a key enzyme in diterpenoid biosynthesis, whereas OS38 transcripts included NAD⁺-dependent farnesol dehydrogenase, associated with sesquiterpene production. Gene Ontology (GO) annotations also confirmed the presence of early pathway enzymes like Acetyl-CoA C-acetyltransferase, essential for initiating the mevalonate pathway. These patterns suggest regulatory flexibility and specialization of terpenoid biosynthesis between the two accessions. Consistent with these results, previous studies in *O. tenuiflorum* reported widespread expression of terpenoid pathway genes, including GGPPS and farnesyl diphosphate synthase^22^. highlighted the significance of Acetyl-CoA C-acetyltransferase in the MVA pathway, aligning with our GO findings. Similarly^36^, demonstrated tissue-specific expression of terpenoid biosynthetic genes in *Withania somnifera*, while^38^ reported that NAD⁺-dependent farnesol dehydrogenase was linked to enhanced sesquiterpene accumulation in Mentha species, comparable to our OS38 observations. The current data support existing evidence of environmentally or developmentally regulated, genotype-specific expression patterns in terpenoid metabolism. These insights hold significance for future applications in metabolic engineering and targeted phytochemical enhancement in medicinal plants.

GC-MS analysis of *O. sanctum* essential oil revealed eugenol as the predominant constituent, followed by β-caryophyllene, β-elemene, and germacrene, underscoring the chemotypic richness and pharmacological relevance of the species. The relative abundance of these compounds varied between the OS1 and OS38 accessions, indicating intraspecific variation in essential oil composition. This chemotypic diversity is important for both therapeutic application and breeding efforts. Similar profiles have been documented in earlier studies. For instance^39^, identified eugenol as the major component in *O. tenuiflorum*, reinforcing its dominance across accessions. Another study^40^ also reported high levels of β-caryophyllene and germacrene in *Ocimum* essential oils, contributing to the plant’s medicinal and aromatic properties. Moreover, the detection of β-elemene, a bioactive sesquiterpene with documented anticancer potential as described by^41^, enhances the therapeutic profile of the oil. The variability in compound proportions between OS1 and OS38 corresponds with earlier observations by^42^, who noted considerable chemotypic diversity within the genus. The use of Adams and NIST spectral libraries ensured accurate compound identification, confirming the presence of key bioactive terpenoids. The findings are consistent with previously reported phytochemical patterns, supporting the reliability of GC-MS-based chemotyping for assessing essential oil variability in *O. sanctum*.

In the present study, relative expression profiling of genes responsible for terpenoid biosynthesis in *O. sanctum* revealed comparatively higher relative expression levels of enzymes such as Eugenol Synthase, Linalool Synthase, Germacrene A Synthase, and Germacrene D Synthase in selected genotypes, indicating transcriptional control as a central mechanism driving terpenoid production. The observed expression patterns were validated by GC-MS analysis, which confirmed the presence of corresponding terpenoid metabolites, thus establishing a functional link between gene expression and metabolite accumulation. These results are in agreement with previous findings by^26^, who reported high expression of eugenol- and linalool-related synthases in *O. tenuiflorum* and *O. basilicum*, correlating with essential oil composition. Similarly^6^, demonstrated that the transcriptional levels of Linalool and Nerolidol Synthases reflected their respective monoterpene concentrations in Arabidopsis and tomato^43^. also showed this relationship in floral scent biosynthesis, reinforcing the model of transcription-metabolite correlation. The expression of Germacrene synthases observed in this study parallels the findings of^5^, who identified their involvement in sesquiterpene diversification in *Ocimum*. No contradictory results were encountered in this analysis; rather, the consistency across gene expression and metabolite profiles emphasizes the transcriptional regulation of secondary metabolism and supports its significance in metabolic engineering strategies for enhancing terpenoid yield in medicinal plants. The comparative transcriptomic observation indicated accession-specific variation in terpenoid biosynthesis between OS1 and OS38 accessions of *O. sanctum*. While both accessions contained transcripts associated with core isoprenoid biosynthetic pathways, OS1 accession showed the presence of a wider range of pathway-associated transcripts, whereas OS38 accession displayed a distinct distribution of terpenoid-related transcripts, including those associated with MVA-linked sesquiterpenoid metabolism. The present findings represent descriptive transcriptomic profiles and should not be interpreted as statistically supported differential expression.

## Conclusion

The high-quality transcriptome data generated from two accessions of *O. sanctum* (OS1 and OS38) provides a robust foundation for understanding terpenoid biosynthesis pathways, with over 333 million quality reads obtained after stringent filtering. The multi-database annotation approach using GO, KEGG has enabled comprehensive functional characterization of the transcripts, leading to the successful identification of numerous trait-specific genes, particularly those involved in terpenoid biosynthesis. The identification of thirty-three distinct terpenoid compounds in the essential oils through GC-MS analysis, coupled with RT-PCR expression profiling of six key genes responsible for terpenoid biosynthesis, provides strong biochemical and molecular evidence supporting the transcriptomic findings. This integrated approach provides transcriptomic insights in to candidate pathways underlying terpenoid production and also established a valuable genomic resource for future breeding programs and biotechnological applications.

This study provides scientific insights into transcript diversity of terpenoid biosynthesis and serves as a basis for future genetic improvement of *O. sanctum*. The transcriptome data generated in this study will serve as a crucial molecular toolkit for researchers working on *O. sanctum* improvement, enabling enhanced genetic diversity assessments, marker development. This work significantly contributes to the molecular understanding of aromatic and medicinal compound biosynthesis in *O. sanctum*, paving the way for improved cultivation strategies and enhanced production of bioactive compounds with therapeutic potential.

## Data Availability

The raw data generated through transcriptome sequencing has been submitted to NCBI SRA database. The SRA ID for OS1 is SRX31706833 and for OS38 is SRX31706834.

## References

[1] Sangwan, N. S., Farooqi, A. H. A., Shabih, F. & Sangwan, R. S. Regulation of essential oil production in plants. *Plant. Growth Regul.* **34**, 3–21 (2001).

[2] Pateraki, I. & Kanellis, A. K. Stress and developmental responses of terpenoid biosynthetic genes in *Cistus creticus* subsp. *creticus*. *Plant. Cell. Rep.* **29**, 629–641 (2010). 20364257 10.1007/s00299-010-0849-1

[3] Hu, T. et al. Identification of biosynthetic pathways involved in flavonoid production in licorice by RNA-seq based transcriptome analysis. *Plant. Growth Regul.* **92**, 15–28 (2020).

[4] Casamassimi, A. et al. Transcriptome profiling in human diseases: new advances and perspectives. *Int. J. Mol. Sci.* **18**, 1652 (2017). 28758927 10.3390/ijms18081652 PMC5578042

[5] Gang, D. R. et al. An investigation of the storage and biosynthesis of phenylpropenes in sweet basil. *Plant. Physiol.* **125**, 539–555 (2001). 11161012 10.1104/pp.125.2.539 PMC64856

[6] Iijima, Y. et al. The biochemical and molecular basis for the divergent patterns in the biosynthesis of terpenes and phenylpropenes in the peltate glands of three cultivars of basil. *Plant. Physiol.* **136**, 3724–3736 (2004). 15516500 10.1104/pp.104.051318 PMC527170

[7] Tripathi, S., Jadaun, J. S., Chandra, M. & Sangwan, N. S. Medicinal plant transcriptomes: the new gateways for accelerated understanding of plant secondary metabolism. *Plant. Genet. Resour.* **14**, 256–269 (2016).

[8] Chandra, M., Kushwaha, S. & Sangwan, N. S. Comparative transcriptome analysis to identify putative genes related to trichome development in *Ocimum* species. *Mol. Biol. Rep.* **47**, 6587–6598 (2020). 32860161 10.1007/s11033-020-05710-1

[9] Chaurasiya, N. D. et al. Withanolide biosynthesis recruits both mevalonate and DOXP pathways of isoprenogenesis in Ashwagandha (*Withania somnifera* L). *Plant. Cell. Rep.* **31**, 1889–1897 (2012). 22733207 10.1007/s00299-012-1302-4

[10] Jadaun, J. S. et al. Over-expression of *DXS* gene enhances terpenoidal secondary metabolite accumulation in rose-scented geranium and *Withania somnifera*. *Physiol. Plant.* **159**, 381–400 (2017). 27580641 10.1111/ppl.12507

[11] Bansal, S. et al. HMG-CoA reductase from camphor tulsi (*Ocimum kilimandscharicum*) regulates MVA-dependent biosynthesis of diverse terpenoids in homologous and heterologous plant systems. *Sci. Rep.* **8**, 3547 (2018). 29476116 10.1038/s41598-017-17153-z PMC5824918

[12] Nagegowda, D. A. Plant volatile terpenoid metabolism: biosynthetic genes, transcriptional regulation and subcellular compartmentation. *FEBS Lett.* **584**, 2965–2973 (2010). 20553718 10.1016/j.febslet.2010.05.045

[13] Gupta, O. P. et al. Contemporary understanding of miRNA-based regulation of secondary metabolites biosynthesis in plants. *Front. Plant. Sci.* **8**, 374 (2017). 28424705 10.3389/fpls.2017.00374 PMC5372812

[14] Sahoo, B. C. et al. Mining of trait specific gene candidates through mRNA sequencing emphasizing on expression study of terpenoid biosynthesis genes in betelvine cash crop. *Ind. Crops Prod.* **162**, 113292 (2021).

[15] Kozarewa, I. & Turner, D. J. 96-plex molecular barcoding for the Illumina Genome Analyzer. *Methods Mol. Biol.* **733**, 279–298 (2011). 21431778 10.1007/978-1-61779-089-8_20

[16] Kircher, M., Sawyer, S. & Meyer, M. Double indexing overcomes inaccuracies in multiplex sequencing on the Illumina platform. *Nucleic Acids Res.* **40**, e3 (2012). 22021376 10.1093/nar/gkr771 PMC3245947

[17] Wang, Z., Gerstein, M. & Snyder, M. RNA-Seq: a revolutionary tool for transcriptomics. *Nat. Rev. Genet.* **10**, 57–63 (2009). 19015660 10.1038/nrg2484 PMC2949280

[18] Lottaz, C., Iseli, C., Jongeneel, C. V. & Bucher, P. Modeling sequencing errors by combining hidden Markov models. *Bioinformatics* **19**, ii103–ii112 (2003). 14534179 10.1093/bioinformatics/btg1067

[19] Adams, R. P. *Identification of essential oil components by gas chromatography/mass spectroscopy* (Allured Publishing Corporation, 2007).

[20] Livak, K. J. & Schmittgen, T. D. Analysis of relative gene expression data using real-time quantitative PCR and the 2 – ∆∆CT method. *Methods* **25**, 402–408 (2001). 11846609 10.1006/meth.2001.1262

[21] Kanehisa, M. et al. KEGG: biological systems database as a model of the real world. *Nucleic Acids Res.* **53**, D672–D677 (2025). 39417505 10.1093/nar/gkae909 PMC11701520

[22] Gupta, P. et al. De novo sequencing and analysis of lemongrass transcriptome provide first insights into the essential oil biosynthesis pathway. *Plant. Cell. Rep.* **36**, 933–945 (2017). 27516768 10.3389/fpls.2016.01129 PMC4963619

[23] Patra, B., Schluttenhofer, C., Wu, Y., Pattanaik, S. & Yuan, L. Transcriptional regulation of secondary metabolite biosynthesis in plants. *Biochim Biophys. Acta Gene Regul. Mech* **1829,** 1236–1247 (2013). 10.1016/j.bbagrm.2013.09.006 24113224

[24] Wang, Q. et al. Metabolic engineering of terpene biosynthesis in plants using a trichome-specific transcription factor MsYABBY5 from spearmint (*Mentha spicata*). *Plant. Biotechnol. J.* **14**, 1619–1632 (2016). 26842602 10.1111/pbi.12525 PMC5067620

[25] Rastogi, S. et al. De novo sequencing and comparative analysis of holy and sweet basil transcriptomes. *BMC Genom.* **15**, 588 (2014). 10.1186/1471-2164-15-588 PMC4125705 25015319

[26] Upadhyay, A. K. et al. Genome sequencing of herb tulsi (*Ocimum tenuiflorum*) unravels key genes behind its strong medicinal properties. *BMC Genom.* **16**, 413 (2015). 10.1186/s12870-015-0562-x PMC4552454 26315624

[27] Bushmanova, E., Antipov, D., Lapidus, A. & Prjibelski, A. D. rnaSPAdes: a de novo transcriptome assembler and its application to RNA-Seq data. *Nat. Methods*. **16**, 265–268 (2019). 10.1093/gigascience/giz100 PMC6736328 31494669

[28] Sudhakar, R. et al. Genome-wide identification and analysis of NAC transcription factor family in *Ocimum tenuiflorum*. *Planta* **251**, 56 (2020).32006110

[29] Singh, A., Dwivedi, U. N. & Shasany, A. K. Genomic analysis of sweet basil (*Ocimum basilicum*) for identification of EST-SSRs and development of genic-SSR markers. *BMC Genom.* **16**, 926 (2015).

[30] Singh, S. et al. Transcriptome analysis in *Withania somnifera* and *Mentha arvensis* reveals key genes involved in terpenoid biosynthesis. *BMC Plant. Biol.* **17**, 302 (2017).

[31] Pandey, A. et al. Transcriptome profiling reveals enriched pathways in *Ocimum kilimandscharicum* and provides insights into secondary metabolite biosynthesis. *BMC Genom.* **22**, 123 (2021).

[32] Suttipanta, N. et al. The transcription factor CrWRKY1 positively regulates the terpenoid indole alkaloid biosynthesis in *Catharanthus roseus*. *Plant. Physiol.* **157**, 2081–2093 (2011). 21988879 10.1104/pp.111.181834 PMC3327198

[33] Van Moerkercke, A. et al. The bHLH transcription factor BIS1 controls the iridoid branch of the monoterpenoid indole alkaloid pathway in Catharanthus roseus. *Proc. Natl. Acad. Sci. USA*. **113**, 12473–12478 (2016). 26080427 10.1073/pnas.1504951112 PMC4491741

[34] Rahimi, M., Mirlohi, A., Saeidi, G. & Arzani, A. Genetic diversity and population structure analysis in basil (*Ocimum basilicum*) using AFLP markers. *Genetika* **50**, 997–1012 (2018).

[35] Kumar, A., Sharma, S. & Mishra, P. Influence of developmental stages and drying methods on essential oil content and composition of sweet basil (*Ocimum basilicum*). *Ind. Crops Prod.* **69**, 484–491 (2015).

[36] Singh, A. et al. Identification of key genes involved in monoterpene biosynthesis and transcriptional regulation in *Ocimum basilicum*. *BMC Plant. Biol.* **20**, 99 (2020).32138663

[37] Thakur, D. et al. Comparative essential oil profile and biological activity of *Ocimum* species: impact of plant tissue differentiation. *3 Biotech.* **6**, 216 (2016).

[38] Rai, A., Saito, K. & Yamazaki, M. Integrated omics analysis of specialized metabolism in medicinal plants. *Front. Plant. Sci.* **12**, 657196 (2021). 10.1111/tpj.13485 28109168

[39] Kothari, S. K., Bhattacharya, A. K. & Ramesh, S. Essential oil yield and quality of methyl eugenol rich *Ocimum tenuiflorum* in relation to plant spacing and harvest time. *Ind. Crops Prod.* **20**, 287–291 (2004). 10.1016/j.chroma.2004.03.019 15553132

[40] Singh, S., Malhotra, M. & Majumdar, D. K. Antibacterial activity of *Ocimum sanctum* fixed oil and linoleic acid. *Phytomedicine* **17**, 110–115 (2010).

[41] Li, N., Wu, X., Zhuang, W., Xiao, Z. & Zhu, D. Triptolide inhibits angiogenesis by targeting the VEGFR2-mediated ERK1/2 signaling pathway. *Cancer Lett.* **319**, 1–11 (2012).22261336

[42] Pushpangadan, P. & Bradu, B. L. Development of phytomedicine and nutraceuticals: needs and prospects. *Curr. Sci.* **69**, 527–533 (1995).

[43] Dudareva, N. et al. E)-β-Ocimene and myrcene synthase genes of floral scent biosynthesis in snapdragon: function and regulation of expression. *Plant. Cell.* **15**, 1227–1241 (2003). 12724546 10.1105/tpc.011015 PMC153728

